# Left ventricular functional recovery of infarcted and remote myocardium after ST-segment elevation myocardial infarction (METOCARD-CNIC randomized clinical trial substudy)

**DOI:** 10.1186/s12968-020-00638-8

**Published:** 2020-06-11

**Authors:** Tomaž Podlesnikar, Gonzalo Pizarro, Rodrigo Fernández-Jiménez, Jose M. Montero-Cabezas, Nina Greif, Javier Sánchez-González, Chiara Bucciarelli-Ducci, Nina Ajmone Marsan, Zlatko Fras, Jeroen J. Bax, Valentin Fuster, Borja Ibáñez, Victoria Delgado

**Affiliations:** 1 Department of Cardiology, Heart Lung Center, Leiden University Medical Center, Albinusdreef 2, 2333 ZA Leiden, The Netherlands; 2 Department of Cardiac Surgery, University Medical Centre Maribor, Maribor, Slovenia; 3 Internal Medicine Clinic, University Medical Centre Ljubljana, Ljubljana, Slovenia; 4 Centro Nacional de Investigaciones Cardiovasculares (CNIC), Madrid, Spain; 5 CIBER de enfermedades CardioVasculares (CIBERCV), Madrid, Spain; 6 Ruber Juan Bravo Hospital Universidad Europea, Madrid, Spain; 7 Zena and Michael A. Wiener Cardiovascular Institute, Icahn School of Medicine at Mount Sinai, New York City, NY USA; 8 Faculty of Medicine University of Maribor, Maribor, Slovenia; 9 Philips Healthcare, Madrid, Spain; 10 Bristol Heart Institute, Bristol NIHR Cardiovascular Research Centre, University of Bristol and University Hospitals Bristol NHS Trust, Bristol, UK; 11 IIS-Fundación Jiménez Díaz University Hospital, Madrid, Spain

**Keywords:** Feature-tracking cardiovascular magnetic resonance, ST-segment elevation myocardial infarction, Intravenous metoprolol, Microvascular obstruction, Intramyocardial hemorrhage, Left ventricular remodeling

## Abstract

**Background:**

We aimed to evaluate the effect of early intravenous metoprolol treatment, microvascular obstruction (MVO), intramyocardial hemorrhage (IMH) and adverse left ventricular (LV) remodeling on the evolution of infarct and remote zone circumferential strain after acute anterior ST-segment elevation myocardial infarction (STEMI) with feature-tracking cardiovascular magnetic resonance (CMR).

**Methods:**

A total of 191 patients with acute anterior STEMI enrolled in the METOCARD-CNIC randomized clinical trial were evaluated. LV infarct zone and remote zone circumferential strain were measured with feature-tracking CMR at 1 week and 6 months after STEMI.

**Results:**

In the overall population, the infarct zone circumferential strain significantly improved from 1 week to 6 months after STEMI (− 8.6 ± 9.0% to − 14.5 ± 8.0%; *P* < 0.001), while no changes in the remote zone strain were observed (− 19.5 ± 5.9% to − 19.2 ± 3.9%; *P* = 0.466). Patients who received early intravenous metoprolol had significantly more preserved infarct zone circumferential strain compared to the controls at 1 week (*P* = 0.038) and at 6 months (*P* = 0.033) after STEMI, while no differences in remote zone strain were observed. The infarct zone circumferential strain was significantly impaired in patients with MVO and IMH compared to those without (*P* < 0.001 at 1 week and 6 months), however it improved between both time points regardless of the presence of MVO or IMH (*P* < 0.001). In patients who developed adverse LV remodeling (defined as ≥ 20% increase in LV end-diastolic volume) remote zone circumferential strain worsened between 1 week and 6 months after STEMI (*P* = 0.036), while in the absence of adverse LV remodeling no significant changes in remote zone strain were observed.

**Conclusions:**

Regional LV circumferential strain with feature-tracking CMR allowed comprehensive evaluation of the sequelae of an acute STEMI treated with primary percutaneous coronary intervention and demonstrated long-lasting cardioprotective effects of early intravenous metoprolol.

**Trial registration:**

ClinicalTrials.gov, NCT01311700. Registered 8 March 2011 - Retrospectively registered.

## Background

Cardiovascular magnetic resonance (CMR) is a powerful noninvasive clinical and research imaging tool for the assessment of the sequelae of an acute myocardial infarction [1]. Within a single scan, assessment of left ventricular (LV) volumes and function, myocardial edema, infarct extent and transmurality, and microvascular damage can be performed. Traditional parameters such as LV volumes and LV ejection fraction and CMR-specific parameters, such as infarct size with late gadolinium enhancement (LGE), presence of microvascular obstruction (MVO) and intramyocardial hemorrhage (IMH) have demonstrated to predict post-infarction LV remodeling and clinical outcome [2–4]. Recently, feature-tracking CMR has been shown to allow for multidirectional myocardial strain assessment from standard CMR cine images without the need for specialized pulse sequences and additional scanning time [5, 6]. Global LV longitudinal strain and circumferential strain with feature-tracking CMR have provided important prognostic information after myocardial infarction [7, 8]. On the other hand, regional LV deformation has been mostly studied in small single-center studies with different CMR tissue tracking techniques, such as myocardial tagging, strain encoded (SENC) imaging, displacement encoding with stimulated echoes (DENSE) imaging or feature-tracking [9–15]. However, the evolution of LV strain after a ST-segment elevation myocardial infarction (STEMI) within infarcted and remote myocardium has not yet been investigated with feature-tracking CMR.

Accordingly, the present sub-analysis of the Effect of Metoprolol in Cardioprotection During an Acute Myocardial Infarction (METOCARD-CNIC) trial [16] evaluated the changes in regional LV peak circumferential strain after STEMI using feature-tracking CMR. The infarct zone and the remote zone circumferential strain were assessed at 1 week and 6 months after STEMI and the effects of the treatment arm (metoprolol versus control), MVO, IMH and adverse LV remodeling on the evolution of infarct and remote zone strain were investigated.

## Methods

### Patient population

The present study included patients who were enrolled in the METOCARD-CNIC trial [16]. Briefly, the multicenter randomized METOCARD-CNIC clinical trial recruited patients with first anterior STEMI undergoing primary percutaneous coronary intervention (PCI). A total of 270 patients were randomized to receive up to 15 mg intravenous metoprolol before reperfusion versus conventional therapy. Of the initial population, 202 patients underwent CMR at 1 week (5 to 7 days) and at 6 months after STEMI. Patients receiving intravenous metoprolol and controls were comparable in terms of demographic characteristics, cardiovascular risk profile, procedural characteristics and discharge medication, as previously described [16–18]. Infarct zone and remote zone circumferential strain were evaluated with feature-tracking CMR at 1 week and 6 months after STEMI. Moreover, the effects of the treatment arm (metoprolol versus control), MVO, IMH and adverse LV remodeling on infarct and remote zone strain were investigated.

The study was approved by the ethical committees and institutional review boards at each participating center. All eligible patients gave written informed consent.

### Cardiovascular magnetic resonance

The CMR protocol has been described in detail elsewhere [19]. Data acquisition was performed with 1.5 and 3 T CMR scanners at 1 week and 6 months after STEMI. LV long-axis views and a stack of contiguous short-axis slices to cover the whole LV were acquired with balanced steady-state free precession (bSSFP) functional cine imaging. Data acquisition parameters were: voxel size 1.6 × 2 mm, slice thickness 8 mm, gap 0 mm, cardiac phases 25–30, TR 3.5, TE 1.7, flip angle 40, SENSE 1.5, averages 1, FOV 360 × 360 mm. Subsequently, edema imaging was performed using a T2-weighted short tau inversion recovery (STIR) sequence, followed by LGE imaging with segmented inversion recovery gradient echo sequence, acquired 10–15 min after intravenous gadolinium contrast agent.

CMR parameters were analyzed with dedicated software (QMass MR 7.5; Medis, Leiden, the Netherlands). LV volumes and function were determined from bSSFP cine short-axis image dataset. Infarct size was defined as the percent of LGE with full-width-half-maximum technique on delayed enhancement images. The presence of MVO, defined as hypointense areas within the hyperenhanced infarct zone, was evaluated by visual assessment on 1-week CMR. Areas with MVO were included in the infarct size. The presence of IMH, defined as hypointense areas within the brighter edematous zone on T2-STIR images, was evaluated by visual assessment on 1-week CMR.

### Feature-tracking cardiovascular magnetic resonance analysis

Feature-tracking CMR analysis was performed with dedicated software (cvi^42^ v5.3, Circle Cardiovascular Imaging, Calgary, Canada). First, the LV endo- and epicardium were delineated in contiguous short-axis slices and the anterior right ventricular insertion point was defined. The most basal slice(s), if the aortic valve plane was present in systolic frames, and most apical slices(s), if myocardial borders were unclear, were excluded. In addition, the mitral annulus and the LV apex were defined in long-axis slices to allow for automated LV segmentation. The outlined myocardium borders were automatically tracked throughout the cardiac cycle with fully automated feature-tracking analysis. The quality of the myocardium tracking was visually evaluated with manual adjustments of the contours if necessary (< 5% of cases). Segmental peak circumferential strain values were obtained according to the 16-segment model of LV [20]. Twenty randomly selected CMR scans were chosen (> 4 weeks after the primary analysis) for the assessment of intra- and inter-observer reproducibility of the segmental circumferential strain measurements.

### Definition of infarct zone and remote zone myocardium

**Fig. 1:**
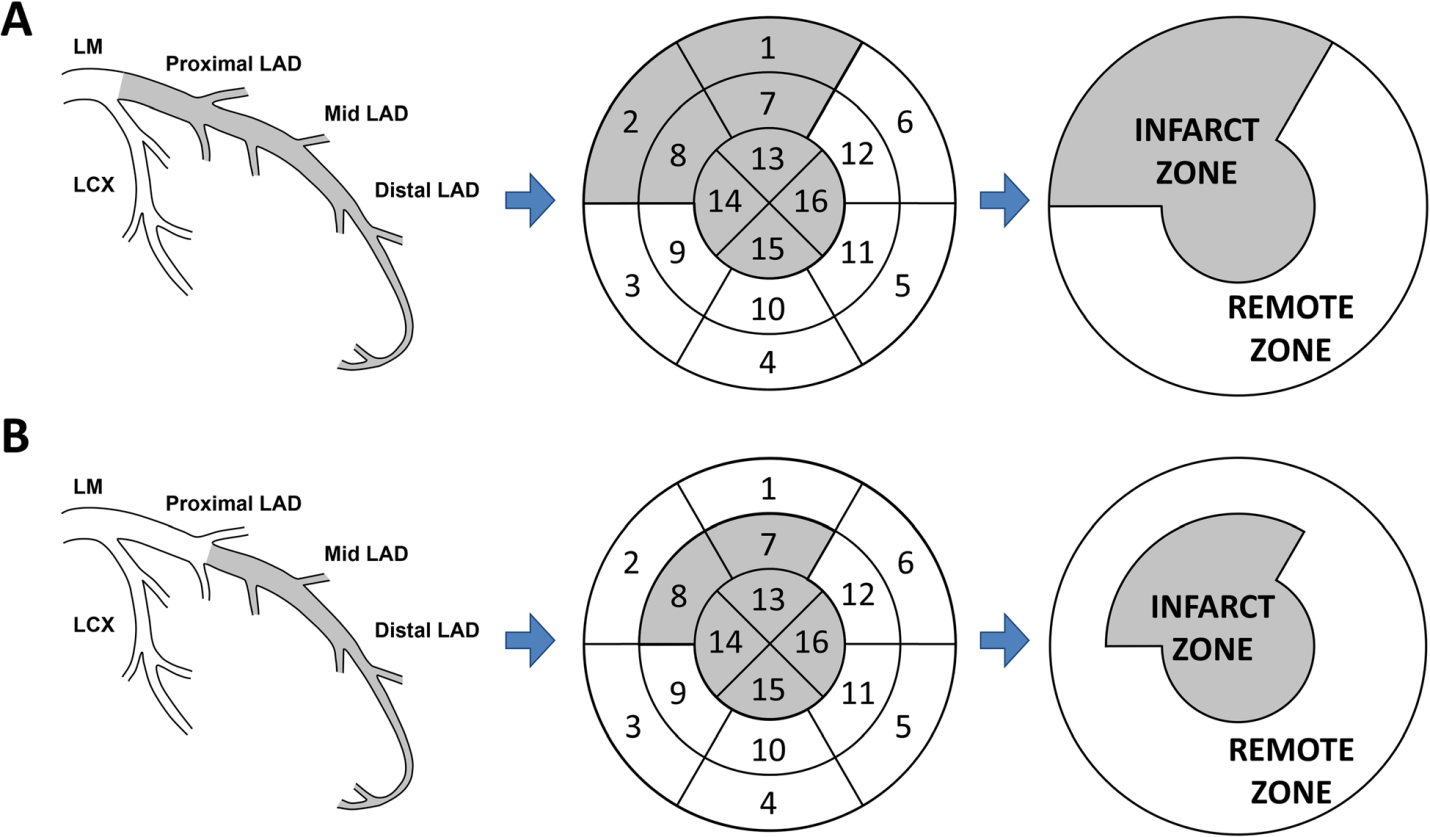
**Definition of the infarct and remote zone myocardium. a**, In case the culprit coronary artery lesion was in the proximal left anterior descending coronary artery (LAD), the infarct zone was defined according to the 16-segment model of the left ventricle (LV) with the segments 1–2, 7–8 and 13–16 and the rest of the LV myocardium was defined as the remote zone. **b**, If the culprit lesion was found in mid or distal LAD, the infarct zone included segments 7–8 and 13–16 and the rest of LV myocardium was defined as the remote zone. LAD, left anterior descending; LCX, left circumflex; LM, left main

The LV myocardium was divided into the infarct zone and the remote zone regions. Taking into consideration that the METOCARD-CNIC trial included a homogeneous population of patients with anterior STEMI and that in > 98% of patients undergoing CMR the culprit lesion was in the left anterior descending coronary artery (LAD) [16], the infarct zone was defined as the LAD perfusion territory. The segmental coronary artery distribution model from the American Society of Echocardiography and the European Association of Cardiovascular Imaging guidelines was used [20]. In addition, according to previous studies with CMR and single-photon emission computed tomography showing that the apical segments most commonly correspond to the LAD perfusion territory, all apical segments were included in the infarct zone [21, 22]. Importantly, the proximal and the mid-distal LAD infarctions were defined differently [23]. When the culprit coronary artery lesion was in the proximal LAD, the infarct zone included the segments 1–2, 7–8 and 13–16 and the rest of LV myocardium was defined as the remote zone (Fig. 1a). If the culprit lesion was in the mid or distal LAD, the infarct zone included the segments 7–8 and 13–16 (the basal anterior and anteroseptal segments were not included), while the rest of LV myocardium was defined as the remote zone (Fig. 1b).

### Study endpoints

The objective of the present analysis was to study the evolution of LV circumferential strain within the infarct zone and the remote zone myocardium in patients with anterior STEMI treated with primary PCI and receiving early intravenous metoprolol versus patients treated with standard of care. Furthermore, the impact of MVO and IMH on infarct and remote zone circumferential strain was investigated. Finally, regional circumferential strain was investigated in patients who developed adverse LV remodeling. Adverse LV remodeling was defined as ≥20% increase in LV end-diastolic volume at 6 months compared to the LV end-diastolic volume at 1 week after STEMI [24]. For each patient population (metoprolol vs. control treatment; presence vs. absence of MVO and IMH; presence vs. absence of adverse LV remodeling) the infarct and remote zone strain values were compared at 1 week and at 6 months after STEMI.

### Statistical analysis

Continuous variables are presented as mean ± standard deviation. Comparisons between 1-week and 6-month infarct and remote zone circumferential strain were performed using paired samples t-test. Comparisons between infarct and remote zone circumferential strain among different groups of patients (with respect to the randomization treatment, the presence of MVO, IMH and adverse LV remodeling) were performed using independent samples t-test. The intra- and inter-observer agreement for the segmental circumferential LV strain measurements were assessed with intraclass correlation coefficients. A two-sided *P*-value of < 0.05 was statistically significant and excellent agreement was defined as an intraclass correlation coefficient > 0.9. All statistical analyses were performed with SPSS (v 23, Statistical Package for the Social Sciences, International Business Machines, Inc., Armonk, New York, USA).

## Results

Of 202 patients with 1-week and 6-month CMR scans, 6 patients were excluded due to a non-LAD infarction (3 patients in the metoprolol group and 3 patients in the control group) [16]. In addition, feature-tracking could not be performed in 5 patients (1 in the early metoprolol group and 4 in the control group) due to CMR image acquisition artefacts. Finally, LV circumferential strain analysis was feasible in 191 patients (early metoprolol group: *N* = 97; control group: *N* = 94) and they formed the population of the present analysis. A proximal LAD infarct was present in 60 patients and a mid-distal LAD infarct was present in 131 patients, with no statistically significant differences between both treatment arms. Patients demographics, cardiovascular risk factors, clinical status at recruitment, procedural characteristics and CMR parameters at 1 week and 6 months after STEMI of the overall population and of the randomization treatment (metoprolol vs control) groups are presented in the Additional file 1: Table 1, and do not differ from those previously published [16–18].

**Table 1:** The effect of early intravenous metoprolol on infarct and remote zone strain

	Infarct zone circumferential strain (%)				Remote zone circumferential strain (%)			
	1 week	6 months	MD [95% CI]	***P***-value*	1 week	6 months	MD [95% CI]	***P***-value*
**Metoprolol (N = 97)**	−9.9 ± 7.6	− 15.7 ± 7.5	−5.7 [− 6.8 to − 4.6]	< 0.001	−19.7 ± 3.6	− 19.2 ± 4.1	0.6 [− 0.2 to 1.3]	0.153
**Control (N = 94)**	−7.2 ± 10.1	−13.2 ± 8.3	−6.0 [− 7.7 to − 4.2]	< 0.001	− 19.3 ± 7.5	− 19.3 ± 3.7	0.0 [− 1.4 to 1.4]	0.984
**MD [95% CI]**	−2.7 [− 5.3 to − 0.1]	−2.5 [− 4.7 to − 0.2]			−0.5 [− 2.1 to 1.2]	0.1 [− 1.0 to 1.2]		
***P*-value**†	0.038	0.033			0.589	0.876		

*CI* confidence interval, *MD* mean difference

*the *P*-values for the strain difference between 6 months and 1 week

†the *P*-values for the strain difference between the group

### Evolution of infarct and remote zone circumferential strain

In the overall population the infarct zone strain significantly improved from 1 week to 6 months after STEMI (from − 8.6% to − 14.5%, mean difference (MD) -5.9%; 95% confidence interval (CI) -6.9 to − 4.8; *P* < 0.001), while no significant changes in the remote zone strain were observed (from − 19.5% to − 19.2%, MD 0.3%; 95% CI − 0.5 to 1.1; *P* = 0.466).

### The effect of early intravenous metoprolol on infarct and remote zone circumferential strain

**Fig. 2:**
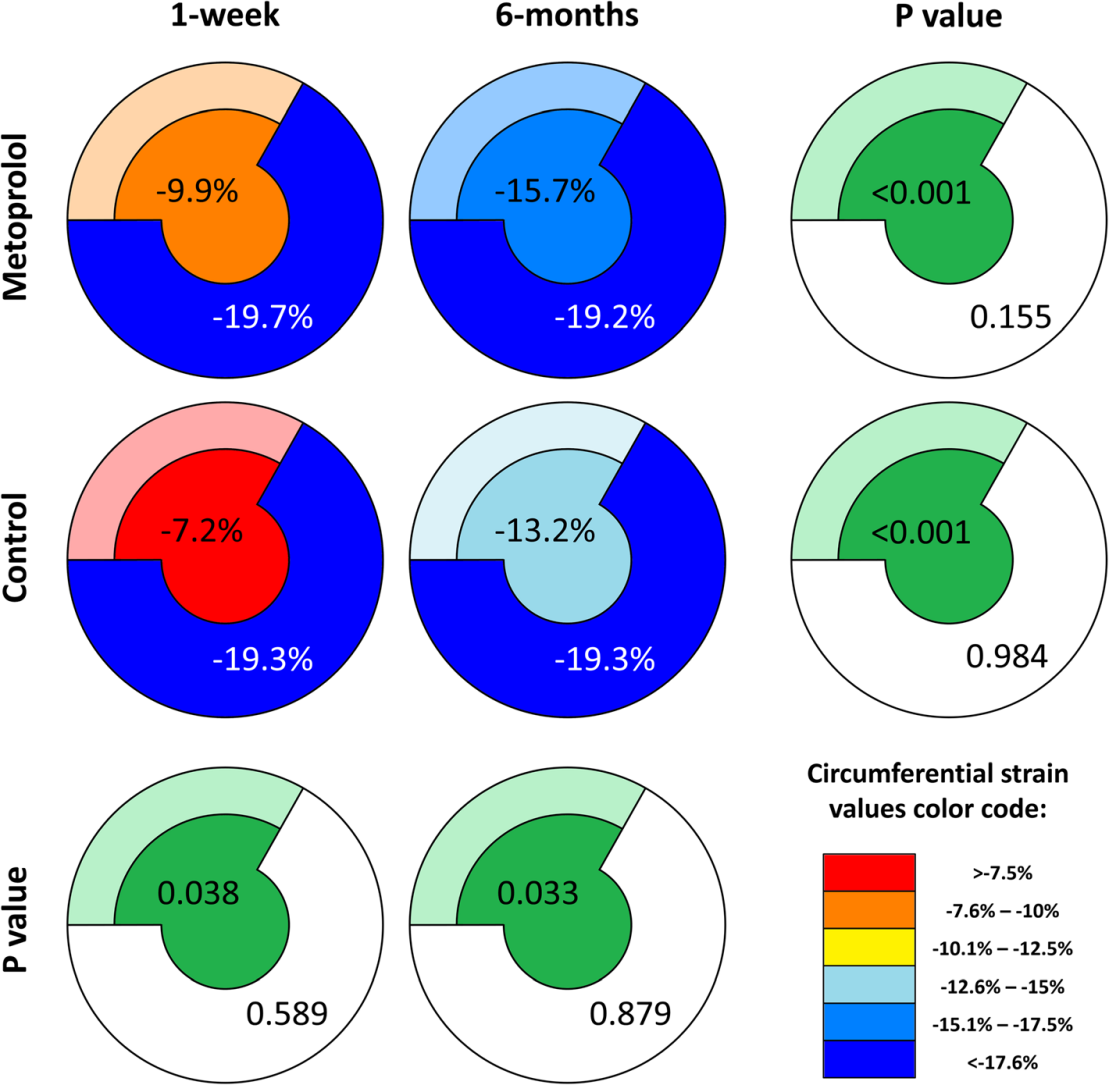
**The infarct zone and the remote zone strain after ST-segment elevation myocardial infarction (STEMI) in patients receiving early intravenous metoprolol versus controls.** Left ventricular (LV) infarct zone and remote zone strains are schematically presented with the mean values in patients receiving early intravenous metoprolol and in patients receiving conventional treatment at 1 week and at 6 months after STEMI. LV was split into the infarct and the remote zone as explained in Fig. 1. In order to schematically present different infarct territories in patients with proximal left anterior descending coronary artery (LAD) coronary artery infarcts and mid-distal LAD infarcts, fainter colors were used to paint the basal anterior and anteroseptal segments, signifying that these segments were either included in the infarct zone (proximal LAD infarcts) or remote zone (mid-distal LAD infarcts) strain analysis. Comparisons between 1-week and 6-month strains are graphically represented on the right-hand side using the same model, with corresponding *P*-values shown separately for the infarct and remote zone strain analysis. In addition, comparisons between the metoprolol and the control group are shown in the bottom row

**Fig. 3:**
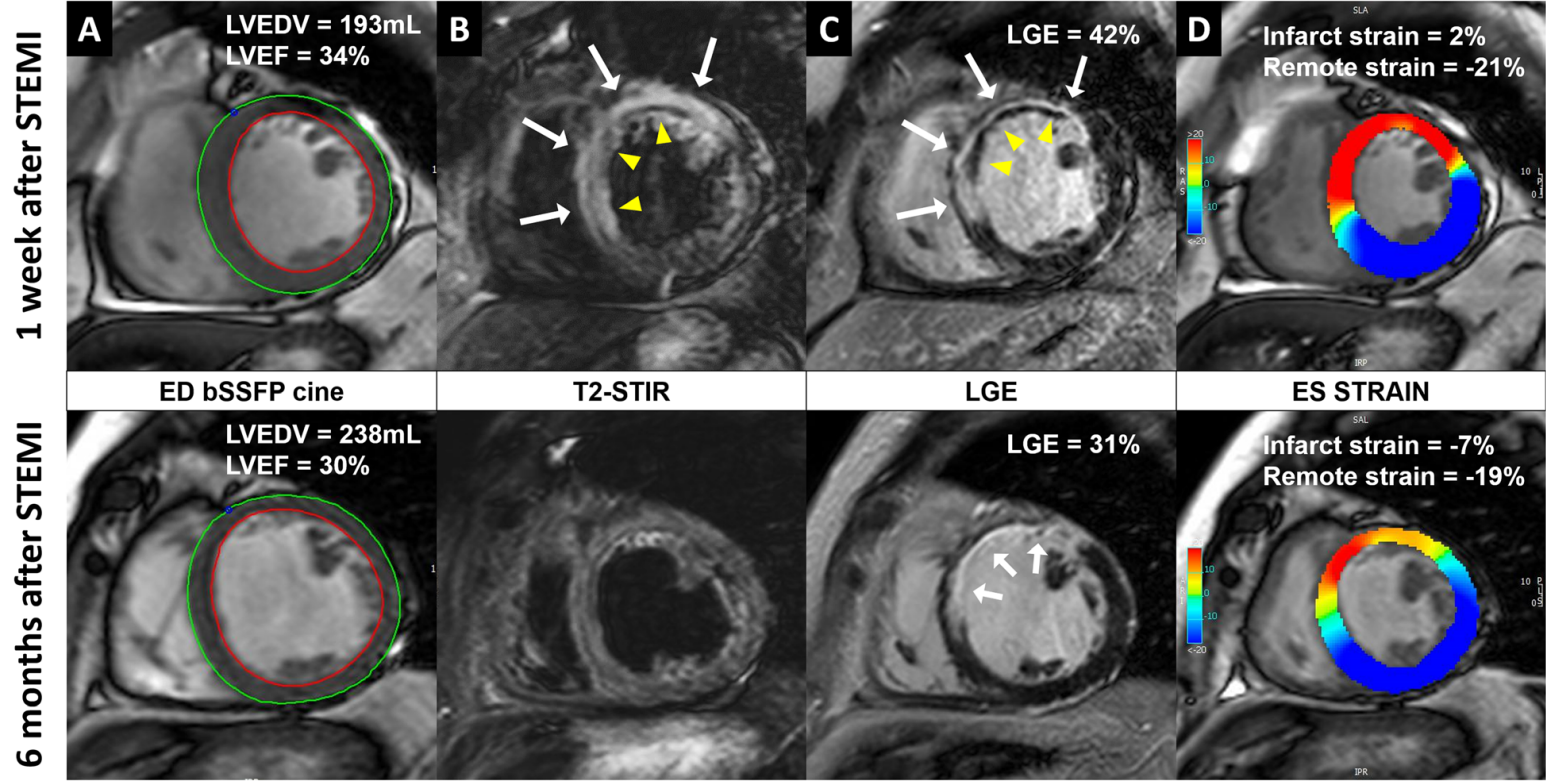
**A patient with a proximal LAD infarct receiving standard STEMI treatment. a**, balanced steady state free precession (bSSFP) end-diastolic images with endo- and epi-cardial contours. The patient developed adverse left ventricular (LV) remodeling (defined as ≥ 20% increase in LV end-diastolic volume (LVEDV) and had a slight reduction in LV ejection fraction (LVEF) at 6 months after STEMI. **b**, T2-weighted short tau inversion recovery (STIR) images, showing the presence of edema (white arrows) and intramyocardial hemorrhage (IMH) (yellow arrowheads) at 1-week after STEMI. **c**, late gadolinium enhancement (LGE) images, showing the presence of acute ischemic injury (white arrows) with microvascular obstruction (MVO, yellow arrowheads) at 1 week after STEMI and infarct scar (white arrows) at 6-months. **d**, end-systolic bSSFP images with feature-tracking circumferential LV strain overlay. At 6 months the infarct zone circumferential strain improved despite the presence of a huge infarct with IMH and MVO in the acute phase, while the remote zone strain slightly declined bSSFP, balanced steady state free precession; ED, end-diastolic; ES, end-systolic; IMH, intramyocardial hemorrhage; LAD, left anterior descending coronary artery, LGE, late gadolinium enhancement; LV, left ventricular; LVEDV, left ventricular end-diastolic volume; LVEF, left ventricular ejection fraction; MVO, microvascular obstruction; SSFP, steady-state free precession; STEMI, ST-segment elevation myocardial infarction; STIR, short tau inversion recovery.

**Fig. 4:**
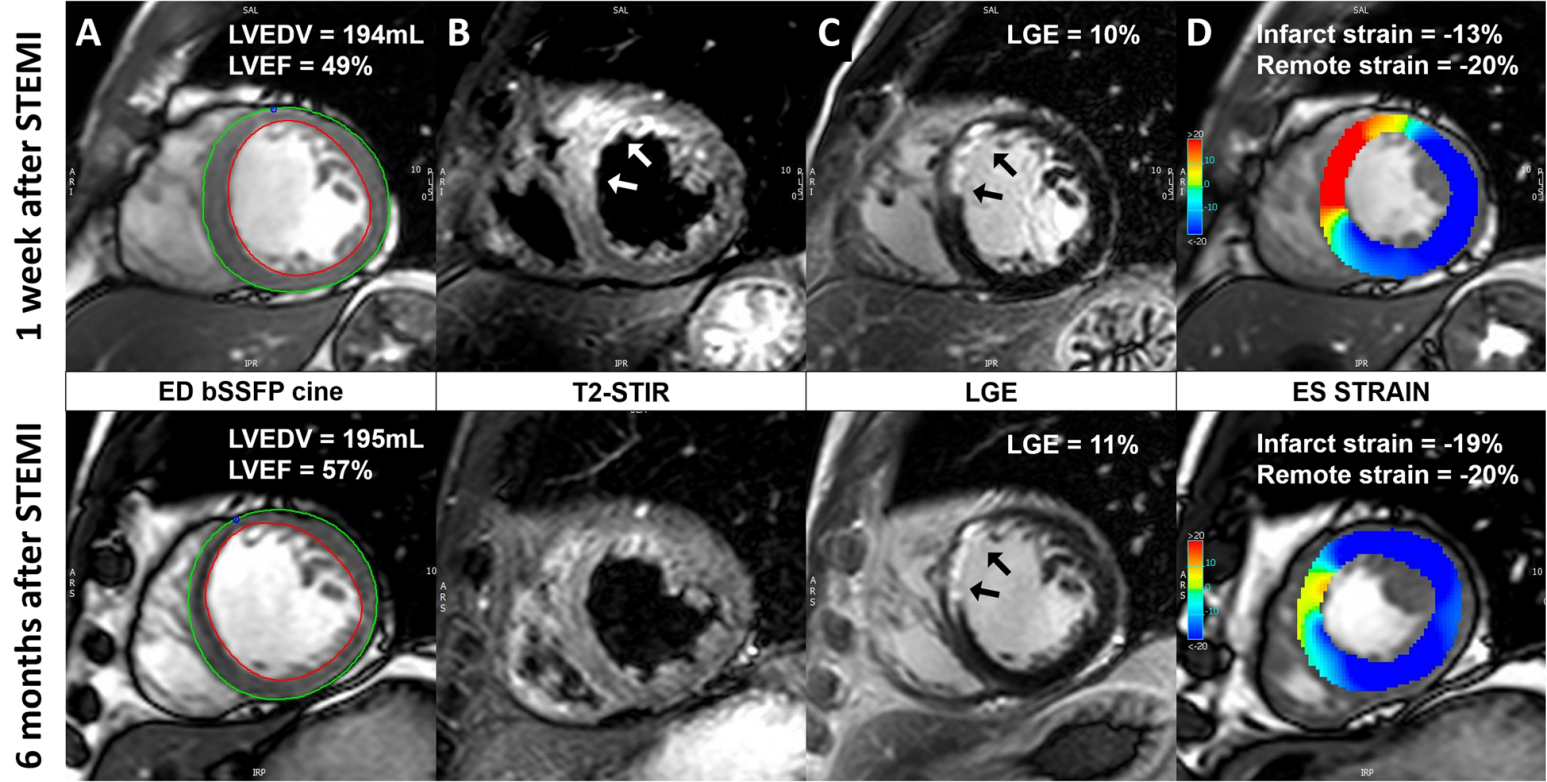
**A patient with a mid-LAD infarct receiving early intravenous metoprolol. a**, bSSFP end-diastolic images with endo- and epi-cardial contours. The LV volumes remained stable and the LVEF increased at 6 months after STEMI. **b**, T2-STIR images showing the presence of edema (white arrows) at 1-week after STEMI. **c**, LGE images showing the presence of acute ischemic injury/infarct scar (black arrows) at 1 week/6-months after STEMI. **d**, end-systolic bSSFP images with feature-tracking circumferential LV strain overlay. At 6 months the infarct zone circumferential strain improved while the remote zone strain remained stable The same abbreviations were used as in Fig. 3

One week after STEMI, patients who received early intravenous metoprolol had more preserved infarct zone strain (*P* = 0.038) compared to the control group, while no significant differences in the remote zone strain were observed (*P* = 0.589). The infarct zone strain significantly improved from 1 week to 6 months after STEMI in both groups of patients (*P* < 0.001), while the remote zone strain remained stable (*P* > 0.05). At 6 months after STEMI, the infarct zone strain remained significantly more preserved among patients receiving early intravenous metoprolol compared to the controls (*P* = 0.033), while no significant differences in the remote zone strain between both treatment arms were observed (*P* = 0.879). The effects of early intravenous metoprolol on infarct and remote zone strain are summarized in Table 1 and Fig. 2. In addition, two patient examples, one receiving early intravenous metoprolol and one receiving standard care, are shown in Figs. 3 and 4.

### The effect of MVO and IMH on infarct and remote zone circumferential strain

The infarct zone strain was significantly more impaired in patients with MVO or IMH, both at 1 week and at 6 months after STEMI (*P* < 0.001). In contrast, there were no differences in remote zone strain between the 2 groups at both time points (*P* > 0.05). Importantly, the infarct zone strain improved from 1 week to 6 months regardless of the presence of MVO and IMH (*P* < 0.001) and remote zone strain remained stable in all groups of patients (*P* > 0.05). The effects of MVO and IMH on infarct and remote zone strain are summarized in Tables 2 and 3. In addition, two patient examples, one with MVO and IMH and one without, are shown in Figs. 3 and 4.

**Table 2:** The effect of microvascular obstruction (MVO) on infarct and remote zone strain

	Infarct zone circumferential strain (%)				Remote zone circumferential strain (%)			
	1 week	6 months	MD [95% CI]	***P***-value*	1 week	6 months	MD [95% CI]	***P***-value*
**MVO (*N*** **= 117)**	− 5.1 ± 8.8	−11.2 ± 7.8	−6.2 [− 7.7 to − 4.7]	< 0.001	− 19.3 ± 7.0	− 19.2 ± 3.8	0.1 [− 1.0 to 1.3]	0.828
**No MVO (*N*** **= 73)**	−14.2 ± 6.1	− 19.5 ± 5.1	− 5.3 [− 6.5 to − 4.1]	< 0.001	− 19.7 ± 3.4	−19.2 ± 4.2	0.5 [− 0.5 to 1.5]	0.313
**MD [95% CI]**	9.2 [6.9 to 11.5]	8.3 [6.5 to 10.2]			0.4 [−1.4 to 2.1]	0.0 [−1.2 to 1.1]		
***P*-value**†	< 0.001	< 0.001			0.677	0.981		

*CI* confidence interval, *MD* mean difference, *MVO* microvascular obstruction

*the *P*-values for the strain difference between 6 months and 1 week

†the *P*-values for the strain difference between the group

**Table 3:** The effect of intramyocardial hemorrhage (IMH) on infarct and remote zone strain

	Infarct zone circumferential strain (%)				Remote zone circumferential strain (%)			
	1 week	6 months	MD [95% CI]	***P***-value*	1 week	6 months	MD [95% CI]	***P***-value*
**IMH (*N*** **= 81)**	−4.1 ± 9.4	−10.5 ± 7.8	−6.4 [−8.3 to − 4.4]	< 0.001	−19.2 ± 7.7	− 19.3 ± 3.7	−0.2 [− 1.7 to 1.4]	0.852
**No IMH (*N*** **= 110)**	− 11.9 ± 7.1	−17.4 ± 6.8	−5.5 [− 6.5 to − 4.4]	< 0.001	−19.8 ± 4.0	− 19.1 ± 4.1	0.6 [− 0.1 to 1.4]	0.102
**MD [95% CI]**	7.8 [5.5 to 10.2]	6.9 [4.8 to 9.0]			0.6 [−1.1 to 2.3]	−0.2 [− 1.3 to 0.9]		
***P*-value**†	< 0.001	< 0.001			0.504	0.736		

*CI* confidence interval, *IMH* intramyocardial hemorrhage, *MD* mean difference

*the *P*-values for the strain difference between 6 months and 1 week

†the *P*-values for the strain difference between the group

### The effect of adverse LV remodeling on infarct and remote zone circumferential strain

There were no statistically significant differences in the infarct zone strain and remote zone strain between patients who did and those who did not develop adverse LV remodeling, both at 1 week and at 6 months after STEMI (*P* > 0.05). Furthermore, in both patient populations the infarct zone strain improved between 1 week and 6 months after STEMI (*P* < 0.001). However, in patients who developed adverse LV remodeling remote zone strain worsened between 1 week and 6 months after STEMI (*P* = 0.036), while in the absence of adverse LV remodeling no significant changes in remote zone strain were observed (*P* = 0.991). The effects of adverse LV remodeling on infarct and remote zone strain are summarized in Table 4. In addition, two patient examples, one with adverse LV remodeling and one without, are shown in Figs. 3 and 4.

**Table 4:** The effect of left ventricular (LV) remodeling on infarct and remote zone strain

	Infarct zone circumferential strain (%)				Remote zone circumferential strain (%)			
	1 week	6 months	MD [95% CI]	***P***-value*	1 week	6 months	MD [95% CI]	***P***-value*
**LV remodeling (*N*** **= 52)**	− 7.6 ± 8.2	−12.6 ± 9.4	−5.0 [−6.6 to − 3.4]	< 0.001	− 20.2 ± 3.7	−19.1 ± 3.7	1.1 [0.1 to 2.1]	0.036
**No LV remodeling (*N*** **= 139)**	−9.0 ± 9.3	−15.1 ± 7.3	−6.2 [− 7.4 to − 4.9]	< 0.001	−19.2 ± 6.5	− 19.3 ± 4.0	0.0 [− 1.0 to 1.0]	0.991
**MD [95% CI]**	1.3 [−1.6 to 4.2]	2.5 [−0.4 to 5.4]			−1.0 [− 2.8 to 0.9]	0.1 [− 1.1 to 1.4]		
***P*-value**†	0.366	0.087			0.317	0.822		

*CI* confidence interval, *LV* left ventricular, *MD* mean difference

*the *P*-values for the strain difference between 6 months and 1 week

†the *P*-values for the strain difference between the group

### Reproducibility of segmental circumferential left ventricular strain measurements

Excellent intra- and inter-observer variabilities for the feature-tracking CMR analysis of the segmental circumferential strain were obtained. The intra-observer intraclass correlation coefficient (95% CI) was 0.925 (0.906–0.940) and the inter-observer intraclass correlation coefficient (95% CI) was 0.907 (0.884–0.926).

## Discussion

The present study shows that the infarct zone circumferential strain improved while the remote zone circumferential strain remained stable between 1 week and 6 months after STEMI. Early intravenous metoprolol had a long-lasting cardioprotective effect on the infarct zone circumferential strain and no significant effect on the remote zone circumferential strain. The infarct zone circumferential strain was significantly impaired in patients with MVO and IMH, but it improved between 1 week and 6 months after STEMI regardless of the presence of MVO or IMH. In patients with adverse LV remodeling, defined as ≥20% increase in LV end-diastolic volume, the infarct zone circumferential strain improved but the remote zone circumferential strain worsened between 1 week and 6 months after STEMI.

### Evolution of the infarct and remote zone circumferential strain

Several studies have used CMR to monitor the evolution of regional LV strain after reperfused myocardial infarction [9–13]. Kidambi et al [9] compared changes in circumferential strain with myocardial tagging in the infarct and remote myocardium in 39 patients who underwent CMR at 2, 30 and 90 days after STEMI. A gradual improvement of the infarct zone circumferential strain was observed (− 10.2, − 16.0% and − 18.6% at days 2, 30 and 90, respectively, *P* < 0.001 for 30-day versus 2-day strain and *P* = 0.04 for 90-day versus 30-day strain) while no significant dynamics in the remote myocardium circumferential strain was observed (− 22.6, − 24.0%, − 24.1% at days 2, 30 and 90, *P* = 0.17 for 90-day versus 2-day strain). Moreover, Gerber et al [11] studied regional circumferential strain with myocardial tagging in 20 patients after myocardial infarction. Myocardial strain improved between day 4 and 7 months in infarcted segments, with no changes observed in the remote myocardium.

Similar to the reported literature, our results demonstrate an improvement in the infarct zone circumferential strain and no significant changes in the remote zone circumferential strain between the acute (1 week) and chronic stage (6 months) of STEMI. However, the present study included a much larger, homogeneous group of patients with anterior STEMI prospectively included in the multi-center randomized controlled clinical METOCARD-CNIC trial [16]. In addition, while other authors have employed different CMR tissue tracking techniques like myocardial tagging, SENC or DENSE imaging in the present analysis LV circumferential strain was evaluated with feature-tracking CMR.

### The effect of early intravenous metoprolol on infarct and remote zone circumferential strain

Early intravenous metoprolol has been associated with improved short-term and long-term outcomes in the METOCARD-CNIC trial [16, 17]. Patients who received intravenous metoprolol prior to primary PCI had significantly reduced infarct size 1 week after STEMI and had more preserved LVEF at 1 week and at 6 months after STEMI. In addition, we have previously shown that patients pre-treated with intravenous metoprolol had more preserved global circumferential strain at 1 week after STEMI, while at 6 months the differences were not significant [18]. However, the present study demonstrates that the infarct zone circumferential strain was more preserved among patients receiving early intravenous metoprolol, both at 1 week and at 6 months after STEMI. This is a very important finding, especially in the view that the differences between the treatment arms in global circumferential strain at 6 months were nonsignificant, underscoring the long-lasting cardioprotective effects of early intravenous metoprolol.

Interestingly, no differences in remote zone circumferential strain were found between patients receiving early intravenous metoprolol and controls. Recently, a slight progressive increase in T2 relaxation time of the remote myocardium has been reported in patients during the first week after STEMI, implying a mild degree of edema of the remote myocardium [25]. Since LV strain is closely associated with post-myocardial infarction edema [9], we may have expected to observe more preserved remote LV circumferential strain in the early intravenous metoprolol group 1 week after STEMI. However, our results imply that the beneficial cardioprotective effects of early intravenous metoprolol were largely confined to the infarct zone myocardium.

### The effect of microvascular obstruction and intramyocardial hemorrhage on infarct zone circumferential strain

MVO and IMH are independent predictors of adverse LV remodeling and clinical outcome after STEMI [3, 4]. However, there is a conflicting evidence on the impact of MVO and IMH on regional strain recovery [10, 12, 13]. Kidambi et al [10] demonstrated an improvement in infarct zone circumferential strain with myocardial tagging between day 2 and day 90 after STEMI in the presence of MVO or IMH. The changes were largely driven by the recovery of epicardial strain (*P* = 0.03 in the presence of MVO and IMH, *P* < 0.01 in the presence of MVO alone), while mid-myocardial and endocardial strain recovery was attenuated (*P* ≥ 0.05). Moreover, O’Regan et al [12] demonstrated a modest improvement of circumferential strain with myocardial tagging in segments with MVO between day 3 and 1 year after STEMI (− 8.1 ± 0.8% and − 14.1 ± 1.0%, respectively; *P* = 0.003). On the other hand, Neizel et al [13] have demonstrated no segmental circumferential strain recovery in the presence of MVO between 3 days and 6 months after STEMI (*P* = 0.2). In our study, the infarct zone circumferential strain was significantly more impaired in patients with MVO or IMH, both at 1 week and 6 months after STEMI, however, it improved between the two time points regardless of the presence of MVO or IMH.

These data indicate the complexity of the healing processes in the infarcted myocardium. Histopathological studies have shown that early infarct tissue consists of a necrotic core, hemorrhage, acute inflammation and islands of tissue repair [26]. These zones often have irregular and patchy distributions and are not confined to the radial location (inner, middle, or outer third) within the infarct [26]. We may reasonably assume that the improvement of infarct zone circumferential strain in patients with MVO and IMH was due to the resorption of edema and necrotic tissue, suggesting a preserved healing capacity of the infarcted myocardium even in the presence of adverse CMR findings.

### The effect of adverse LV remodeling on remote zone circumferential strain

Similar to the majority of previous studies [9, 11] we have shown no differences in the evolution of remote zone circumferential strain in the overall population, as well as in patients divided according to the randomization treatment, patients with MVO and IMH. However, patients with adverse LV remodeling presented with a small, but statistically significant worsening of the remote zone circumferential strain. Bulluck et al [27] demonstrated increased extracellular volume fraction of the remote myocardium acutely and at 5 ± 2 months after STEMI in patients who developed adverse LV remodeling (defined as ≥20% increase in LV end-diastolic volume). Moreover, remote zone noncontrast T1 mapping provided independent and incremental prognostic information above the clinical risk factors and traditional CMR outcome markers in STEMI patients treated by primary PCI [28]. These findings indicate that LV remodeling after myocardial infarction is a complex and multifactorial process that may involve excessive inflammation/fibrosis of the remote myocardium and may result in impaired circumferential strain.

### Limitations

Feature-tracking is a novel CMR technique to assess LV strain. Reference values for LV strain and the agreement between different vendors of feature-tracking software are largely unknown [29]. Furthermore, evaluation of LV strain was not a predefined study endpoint of the METOCARD-CNIC trial. Of the initial 196 patients with a LAD infarct who underwent 2 CMR studies in the METOCARD-CNIC trial, 5 patients were excluded from the LV strain analysis due to poor CMR cine image quality, which may have influenced our results. However, 97% feasibility of strain assessment with feature-tracking CMR is similar to what has been described previously [30, 31], and excellent intra- and inter-observer reproducibility of the segmental feature-tracking strain analysis were observed, similar or slightly better to what has been reported in the literature [32, 33]. In the present analysis we did not analyze regional radial and longitudinal strain since previous studies have shown that, on the segmental level, feature-tracking-derived circumferential strain is the most robust and has the lowest intra- and inter-observer variability [30, 31].

## Conclusion

Regional LV circumferential strain analysis with feature-tracking CMR has revealed several important insights on the impact of MVO, IMH and adverse LV remodeling on the evolution of the infarct zone and remote zone circumferential strain. Furthermore, in patients with first anterior STEMI treated with primary PCI long-lasting cardioprotective effects of early intravenous metoprolol treatment on the infarct zone strain were demonstrated.

## Supplementary information

**Additional File 1.** Table 1: Patients demographics, cardiovascular risk factors, clinical status at recruitment, procedural characteristics and CMR parameters at 1 week and 6 months after STEMI

## Data Availability

The datasets used and analyzed during the current study are available from the corresponding author on reasonable request.

## Abbreviations

bSSFP: Balanced steady state free precession
CMR: Cardiovascular magnetic resonance
DENSE: Displacement encoding with stimulated echoes
IMH: Intramyocardial hemorrhage
LAD: Left anterior descending coronary artery
LCX: left circumflex coronary artery
LGE: Late gadolinium enhancement
LM: Left main coronary artery
LV: Left ventricle/left ventricular
METOCARD-CNIC: Effect of Metoprolol in Cardioprotetion During an Acute Myocardial Infarction
MVO: Microvascular obstruction
PCI: Percutaneous coronary intervention
SENC: Strain encoded
STEMI: ST-segment elevation myocardial infarction
STIR: Short tau inversion recovery
