# Supplementary material for: Left ventricular functional recovery of infarcted and remote myocardium after ST-segment elevation myocardial infarction (METOCARD-CNIC randomized clinical trial substudy)
Source: J Cardiovasc Magn Reson. 2020 Jun 11;22:44. doi: 10.1186/s12968-020-00638-8 (PMC7288440; doi:10.1186/s12968-020-00638-8)
Supplement: Supplementary file 1 — Additional File 1. Table 1: Patients demographics, cardiovascular risk factors, clinical status at recruitment, procedural characteristics and CMR parameters at 1 week and 6 months after STEMI [file 12968_2020_638_MOESM1_ESM.docx]

**Table 1: Patients demographics, cardiovascular risk factors, clinical status at recruitment, procedural characteristics and CMR parameters at 1 week and 6 months after STEMI**

|  | **Total**  **(N=191)** | **Metoprolol**  **(N=97)** | **Control**  **(N=94)** | **p-value** |
| --- | --- | --- | --- | --- |
| **Demographics** | | | | |
| Age (years) | 57.9±11.2 | 57.4±12.2 | 58.4±10.2 | 0.539 |
| Sex (male) | 168 (88) | 83 (88) | 85 (88) | 0.887 |
| BMI (kg/m^2^) | 27.6±3.5 | 27.6±3.5 | 27.6±3.6 | 0.964 |
| **Cardiovascular risk factors** | | | | |
| Hypertension | 72 (38) | 35 (36) | 37 (39) | 0.680 |
| Diabetes mellitus | 39 (20) | 21 (22) | 18 (19) | 0.642 |
| Dyslipidemia | 83 (44) | 42 (43) | 41 (44) | 0.985 |
| Smoking* | 124 (65) | 64 (66) | 60 (64) | 0.681 |
| **Clinical status at recruitment** | | | | |
| Killip class II^†^ | 19 (10) | 8 (8) | 11 (12) | 0.425 |
| Systolic BP (mmHg) | 142±19 | 142±18 | 143±20 | 0.892 |
| Diastolic BP (mmHg) | 88±16 | 90±16 | 87±16 | 0.246 |
| Heart rate (bpm) | 82±13 | 82±14 | 81±13 | 0.777 |
| **Procedural characteristics** | | | | |
| Ischemia duration (min) | 194±65 | 198±63 | 190±67 | 0.354 |
| TIMI grade 0-1 flow before primary PCI | 158 (83) | 77 (79) | 81 (86) | 0.272 |
| Successful PCI (TIMI grade 2-3 flow) | 188 (98) | 97 (100) | 91 (97) | 0.117 |
| **CMR parameters at 1 week** | | | | |
| LVEDV (mL) | 173.1±36.2 | 170.8±33.4 | 175.4±38.9 | 0.378 |
| LVESV (mL) | 98.4±31.4 | 93.6±26.8 | 103.3±35.1 | **0.032** |
| LVEF (%) | 44.0±25.5 | 45.7±9.2 | 42.3±9.5 | **0.012** |
| LV mass (g) | 111.8±25.5 | 109.9±25.2 | 113.8±25.8 | 0.287 |
| LGE (%) | 23.0±12.9 | 21.1±11.7 | 25.0±13.8 | **0.036** |
| MVO | 117 (61) | 52 (54) | 65 (69) | **0.034** |
| IMH | 81 (42) | 36 (37) | 45 (48) | 0.133 |
| LV GCS (%) | -13.2±3.9 | 13.9±3.8 | 12.5±3.9 | **0.011** |
| **CMR parameters at 6 months** | | | | |
| LVEDV (mL) | 192.3±43.2 | 187.4±39.0 | 197.4±46.8 | 0.112 |
| LVESV (mL) | 105.4±41.3 | 98.5±36.3 | 112.5±45.1 | **0.020** |
| LVEF (%) | 46.7±10.9 | 48.6±10.1 | 44.7±11.4 | **0.013** |
| LV mass (g) | 85.8±17.8 | 84.8±17.6 | 86.9±18.0 | 0.426 |
| LGE (%) | 17.0±9.8 | 15.8±9.7 | 18.2±9.8 | 0.099 |
| Adverse LV remodeling^‡^ | 52 (27) | 21 (22) | 31 (33) | 0.079 |
| LV GCS (%) | -16.4±4.3 | 16.9±4.1 | 15.8±4.4 | 0.087 |

BMI, body mass index; BP, blood pressure; CMR, cardiovascular magnetic resonance; GCS, global circumferential strain; IMH, intramyocardial hemorrhage; LGE, late gadolinium enhancement; LV, left ventricular; LVEDV, left ventricular end-diastolic volume; LVEF, left ventricular ejection fraction; LVESV, left ventricular end-systolic volume; MVO, microvascular obstruction; PCI, percutaneous coronary intervention; STEMI, ST-segment elevation myocardial infarction; TIMI, Thrombolysis in Myocardial Infarction.

*smoking was defined as current or quitted <10 years ago

†all other patients were Killip class I (Killip class III to IV were study’s exclusion criteria)

‡adverse LV remodeling was defined as ≥20% increase in LVEDV at 6 months

Continuous variables are presented as mean ± standard deviation and categorical variables as frequencies (percentages). Comparisons between the early metoprolol group and the control group were performed using independent samples t-test for continuous variables and Pearson’s Chi square test or Fischer’s exact test for categorical variables. Fischer’s exact test was used when the expected value of a categorical variable was <5.
